# Influence of Accelerated Aging on the Fiber-Matrix Adhesion of Regenerated Cellulose Fiber-Reinforced Bio-Polyamide

**DOI:** 10.3390/polym15071606

**Published:** 2023-03-23

**Authors:** Celia Katharina Falkenreck, Nicole Gemmeke, Jan-Christoph Zarges, Hans-Peter Heim

**Affiliations:** Institute of Material Engineering, Polymer Engineering, University of Kassel, Moenchebergstr. 3, 34125 Kassel, Germany

**Keywords:** resistance, cellulose, fiber reinforcement, bio-polyamide, temperature, hydrolysis, mechanical properties, automotive

## Abstract

With regard to the sustainability and biological origin of plastic components, regenerated cellulose fiber (RCF)-reinforced polymers are expected to replace other composites in the future. For use under severe conditions, for example, as a housing in the engine compartment, the resistance of the composites and the impact on the fiber and fiber-matrix adhesion must be investigated. Composites of bio-polyamide with a reinforcement of 20 wt.% RCF were compounded using a twin-screw extruder. The test specimens were manufactured with an injection molding machine and aged under conditions of high humidity at 90% r. H, a high temperature of 70 °C, and water storage using a water temperature of 23 °C for 504 h. Mechanical tests, single-fiber tensile tests (SFTT), single-fibre pull-out tests (SFPT), and optical characterization revealed significant changes in the properties of the composites. The results of the SFPT show that accelerated aging had a significant effect on the bio-polymer and an even stronger effect on the fiber, as the single-fiber tensile strength decreased by 27.5%. Supplementary notched impact strength tests revealed a correlation of the impact strength and the accelerated aging of the RCF-reinforced composites. In addition, it could be verified that the tensile strength also decreased at about 37% due to the aging effect on the RCF and a lowered fiber-matrix adhesion. The largest aging impact was on the Young’s modulus with a decrease of 45% due to the accelerated aging. In summary, the results show that the strengthening effect with 20 wt.% RCF was highly decreased subsequent to the accelerated aging due to hydrolysis and debonding because of the shrinkage and swelling of the matrix and fiber. These scientific findings are essential, as it is important to ensure that this bio-based material used in the automotive sector can withstand these stresses without severe degradation. This study provides information about the aging behavior of RCF-reinforced bio-based polyamide, which provides fundamental insights for future research.

## 1. Introduction

Polysaccharide cellulose is an almost inexhaustible raw material with fascinating structural potential. It is characterized by hydrophilicity, biodegradability, flexibility, and a broad chemical modifiability [1]. Using the chemical viscose process, the cellulose can be improved chemically and structurally without losing the advantages of the raw material. The result is regenerated cellulose fiber, also called viscose or human-made cellulose [2]. Although the viscose process is chemically critical, especially in comparison to the production process of lyocell fibers, RCFs are distinguished by a higher ductility and higher thermal resistance compared to their natural counterparts and other human-made cellulose fibers [3]. These properties support a high tensile strength, as the fibers do not break as easily during the manufacturing process, which results in a longer fiber length [4]. In addition, the uniform shape, surface quality, and the chemical structure of the cellulose ensures great fiber-matrix adhesion. In perspective, RCFs can be produced in large industrial quantities to be used as an alternative for glass fibers in the automotive industry.

For more than ten years, research has been conducted on climate-friendly composites in order to cope with the high ecological demands of mobility. They are not only an alternative to petro-based polymers as the matrix, but bio-based fibers can also contribute to the CO_2_-reduced production of components. Especially in the automotive sector, in addition to greenhouse gas reduction, the enhancement of the lightweight potential is of high interest [5]. The latter can be achieved mainly due to the low density of cellulose-based fibers in comparison to glass fibers [6]. In addition, several bio-polymers have a lower density, and these contribute to the increased lightweight potential as well [5]. However, their mechanical properties and their resistance against environmental factors also have to be considered to ensure their ability to meet the automotive standards, and hence, to compete with the composites used conventionally. Furthermore, mass production with the new bio-based materials must be possible at a low cost as well, which is currently rather difficult due to the high material costs of bio-based polymers [7,8].

Unlike other bio-polymers, such as polylactide (PLA), bio-polyamides belong to the engineering polymers. For this reason, they can be used for highly stressed components or under severe environmental conditions [9]. In order for these composites to be accepted as an alternative in the industry, the resistance of the bio-polyamides must be investigated. Several studies have already examined cellulose fiber-reinforced bio-based polyamides, e.g., PA4.10, PA6.10, PA10.10, and PA11. They have shown significant increases in all mechanical properties, especially that the notched impact strength increases depending on the fiber content [10,11,12]. In addition, the relationship between the fiber-matrix adhesion and the mechanical properties has also been addressed in different studies [13,14,15,16]. In this study, a comparatively new bio-based polyamide was investigated—PA5.10. Compared to conventional PA6, PA5.10 is characterized by a lower water absorption and a high oxygen barrier, with similar mechanical properties [17]. It is produced by combining microbial fermentation for the production of cadaverine and the polymerization of bio-based cadaverine with sebacic acid [18,19].

It is well known that polyamides and cellulose fibers (separate consideration) absorb a lot of moisture when exposed to high humidity or fluids due to their hygroscopic properties. Furthermore, high temperatures accelerate the absorption and the resulting processes of hydrolysis [20]. This circumstance can be explained by Fickian diffusion, which shows a rapid uptake of liquid due to high temperatures. This results in a weight increase and accelerated swelling of the matrix and the fibers, which leads to cracks all over the matrix [21,22]. Hydrolysis itself leads to shortened chains and a degradation of the polymer due to the splitting processes of the microstructure [23]. These phenomena have to be included in the consideration of the accelerated aging of polymers.

In this study, composites for the automotive sector were investigated. The aim was to develop materials for the use of mechanically and thermally highly stressed components, for example, in the engine compartment or as battery housings. To ensure the suitability and durability of the compound, and in particular, the fiber-matrix adhesion, defined accelerated aging conditions were developed. Test specimens were stored under environmental conditions including high temperatures, high humidity, and water storage. The aim was to determine how resistant the compound is against hydrolysis and thermal degradation.

In this study, the composites were evaluated with the use of mechanical and optical tests. The main focus was the consideration of the fiber-matrix adhesion and its correlation with accelerated aging. With single-fiber tensile and single-fiber pull-out tests, the interfacial shear strength and the critical fiber length of the RCF were ascertained. A correlation between the fiber and the integral mechanical properties of the composites was determined. The considerations of the damaging effect on the RCF due to artificial aging and the influence on the mechanical properties of fiber-reinforced bio-based PA composites are novel and add value to the research on bio-based composite alternatives. The consideration of long-term durability has not yet been investigated with bio-polyamides in this context, and this study introduces a new field of research related to fiber-matrix adhesion.

## 2. Materials and Methods

### 2.1. Fiber and Matrix

For the investigations, the 100% bio-based polyamide AKROMID^®^ NEXT 5.10 3 EXP nature (PA5.10) from AKRO-PLASTIC GmbH (Niederzissen, Germany) was used. The bio-based raw material is obtained from corn and castor oil. It has a density of 1.07 g/ cm³ and a melting temperature of 217 °C [18,24]. In order to be able to compare the impact of the fiber, both fiber-reinforced (20 wt.%) and non-reinforced samples were analyzed. The fiber reinforcement was implemented using raw white regenerated cellulose fiber (RCF) named rayon from Cordenka GmbH & Co. KG (Obernburg am Main, Germany). Bio-based and biodegradable RCF is human-made using the viscose process [2]. It features a density of 1.5 g/cm^3^, a fiber diameter in the range of 12–15 µm, a fiber tensile strength of 830 MPa and a fineness of 1.85 dtex [25]. The resulting continuous filaments are chopped to a length of 2.5 mm.

### 2.2. Accelerated Aging

Accelerated aging was performed to evaluate the durability, and in particular, the mechanical resistance of the fiber-reinforced and non-reinforced bio-polyamide PA5.10. Test specimens were aged under the conditions of high humidity (H) of 90% r. H. and 23 °C, a high temperature (T) of 70 °C and 50% r. H., and water storage (W) using a water temperature of 23 °C. The reference value was stored in a standard climate (S) of 23 °C and 50% r. H. according to DIN EN ISO 291 [26]. Aging with high temperature and high humidity was carried out using the ATLAS SolarClimatic SC340 MHG climatic chamber from Weiss Technik GmbH (Reiskirchen, Germany).

Accelerated aging was carried out for 504 hours. The following composites in Table 1 resulted from these climate storage scenarios.

### 2.3. Preparation of the Composite

A composite based on the bio-based PA5.10 with a fiber content of 20 wt.% RCF was prepared and examined in this study. No additives or coupling agents were used.

**Figure 1:**
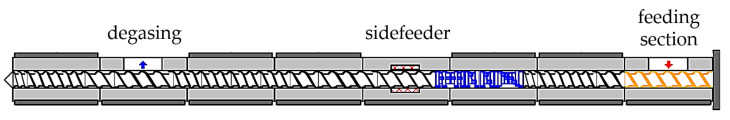
Configuration of the twin screws.

The composite was prepared using the ZSE 18 HPe twin-screw extruder from Leistritz Extrusionstechnik GmbH (Nuremberg, Germany). The screw diameters measured 18 mm, with a process length of 40D.The screw configuration is shown in Figure 1. The section in front of the sidefeeder consisted of kneading blocks and conveying elements with an enlarged free volume. In order to not damage the fibers, after the addition by the sidefeeder, only conveying elements were arranged. To ensure the dryness of the granules prior to processing, the PA5.10 was dried to below a 0.1% moisture content in the Dry Jet Easy dry air dryer from TORO-Systems (Igensdorf, Germany) at 80 °C for four hours, as recommended by Acierno and van Puyvelde [27]. The RCFs were dried using an air convection oven at 105 °C for 24 h until the moisture content was lowered to 0.5%.

PA5.10 granules were fed into the feeding section of the twin-screw extruder, while the chopped fibers were added later in the process using the sidefeeder. The quantity shares were measured with gravimetric scales, which are included in the feeding system from Brabender Technology GmbH & Co. KG (Duisburg, Germany). The measured melt temperature at the nozzle was approx. 240 °C, and the melt pressure was within a range of 40–65 bar. The minimum processing temperatures were selected so that the RCFs, which degrade above 200 °C, were as least damaged as possible while the matrix remained flowable [25,28]. The used temperature profile of the twin-screw extruder is shown in Table 2.

After the compounding, the composite strand was cooled down with compressed air on a discharge conveyor and pelletized with a Scheer SGS 25.E from Maag Germany GmbH (Grossostheim, Germany) to a length of 3–4 mm.

### 2.4. Injection Molding

All material tests carried out in this study required standardized testing. Thus, type 1A test specimens according to DIN EN ISO 527-2 were prepared using the Allrounder 320C injection molding machine from Arburg GmbH & Co. KG (Lossburg, Germany). The machine features a clamping force of 500 kN and a screw diameter of 25 mm. Similar to the compounding process, the composite was dried for four hours at 80 °C to below a moisture content of 0.1% to avoid residual moisture in the granules. 

The temperature profiles of the injection molding processes can be seen in Table 3. All injection molded components were produced with a cold runner. Non-reinforced test specimens and test specimens with a fiber content of 20 wt.% were prepared. The melt temperature of the fiber-reinforced granules was set to 240 °C to reduce the thermal degradation of the RCF. Overall, the cycle time was approx. 70 s, including a packing time of 21 s at 550 bar and a cooling time of 35 s in both injection molding processes. The injection pressure of the process without fibers was within a range of 350–370 bar, while the pressure was increased to 1400–1550 bar for the fiber-reinforced composites. 

### 2.5. Fiber Length Distribution 

The fiber length distribution of the raw, compounded, and injection-molded specimens were measured using the QICPIC dynamic image analysis system from Sympatech GmbH (Clausthal-Zellerfeld, Germany). The procedure works with the MIXCEL liquid dispersion unit, which uses isopropanol as the carrier medium of the fibers. The fibers were previously extracted from the matrix using a simplified soxhlet extraction [29]. Small pieces of the parallel section of a 1A test specimen and highly concentrated (98%) formic acid were placed into a beaker at 23 °C for 5 h to dissolve the bio-polyamide matrix.

Subsequently, the fibers are put into the liquid dispersion unit and transported through a 2 mm cuvette past a high-speed camera in the isopropanol flow. The camera then takes images of each fiber. Per test, approx. 80,000 fibers were captured by the camera at a frequency of 60 Hz. The M7 objective was chosen because a range of fiber lengths of 12.6 µm–8.66 mm can be observed with a high resolution of 4.2 µm. Sympatech software WINDOX calculated the length of the passed fibers and created a number-based distribution function q0.

### 2.6. Single-Fiber Tensile Test (SFTT)

Prior to the single-fiber pull-out tests, the tensile properties of the fibers were investigated. Single-fiber tensile tests were carried out using the FAVIMAT+ from Textechno Herbert Stein GmbH & Co. KG (Moenchengladbach, Germany), at a speed of 5 mm/min. A preload of 0.5 cN/dtex was used to implement the same setting parameters as in the SFPT. The RCFs were tested after three weeks in a standard climate, in high temperature and humidity, and in water storage. Ten fibers per aging condition with a free clamping length of 20 mm were tested. 

### 2.7. Single-Fiber Pull-Out Test (SFPT)

To determine the influence of the accelerated aging on the fiber-matrix adhesion, SFPT were carried out using raw RCF with 504 hours aged (T, H, W) PA5.10 as well as using PA5.10 at standard climate with 504 hours aged (T, H, W) fibers. The SFPT is a method to determine the interfacial shear strength and the critical fiber length of the RCF. The test has also already been carried out by Zarges et al. [13] and Graupner et al. [30]. The required equations by Kelly and Tyson can be seen below [31]. Equation (1) calculates the interfacial shear strength (IFSS) by dividing the maximum Force *F_max_* of the pull-out with the diameter of the RCF *d_f_* and the embedded fiber length *l_ef_*. The second equation determines the critical fiber length using the calculated IFSS and the tensile strength of the RCF *σ_f_*. The latter has to be ascertained first using the single-fiber tensile test.

(1) $$\tau =\frac{F_{max}}{d_{f}\times \pi \times l_{ef}}$$

(2) $$l_{c}=\frac{\sigma_{f}\times d_{f}}{2\times \tau }$$

Both equations require an equal fiber diameter, a uniform stress distribution, and a homogeneous embedding of the fiber in the matrix. Only if these requirements are met, the force, which is required to pull out the fiber, creates a linear relation with the embedded fiber length. Since it is unlikely that all conditions will be met, the results are designated as the apparent interfacial shear strength and the apparent critical fiber length [31].

**Figure 2:**
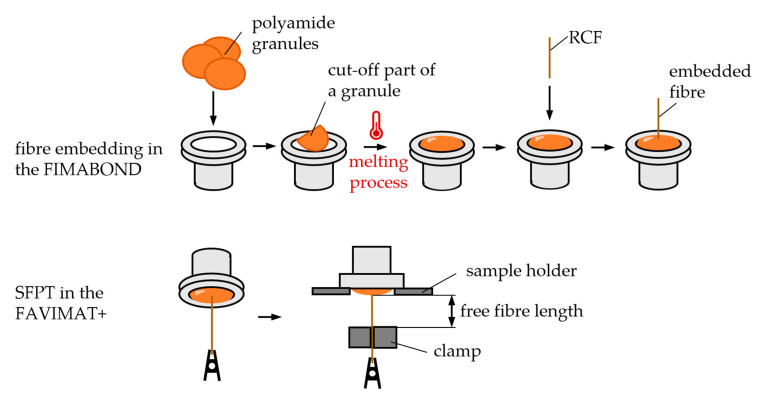
Illustration of the embedding process and pull-out testing.

**Figure 3:**
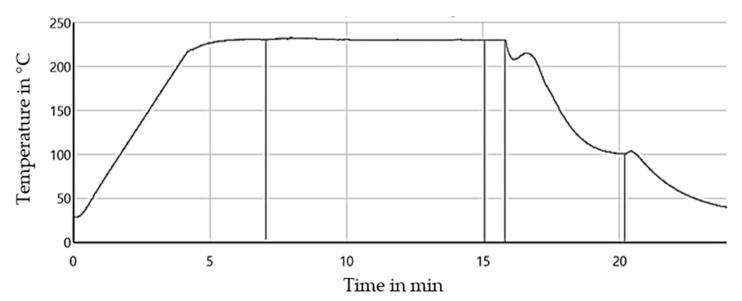
Temperature–time curve of the embedding process using the Fimabond.

Before the test can be carried out, the sample has to be prepared. The preparation and pull-out process can be seen in Figure 2. The preparation process was carried out with the FIMABOND system from Textechno Herbert Stein GmbH & Co. KG (Moenchengladbach, Germany). A granule or small amount of the parallel section of an aged test specimen (see Section 2.4) was cut and placed in a crucible. Heating at a temperature of 230 °C melted the small piece of polymer and a single RCF was automatically inserted into the molten matrix by the machine. An embedding speed of 500 µm/min and an embedding depth of 350 µm was used. Subsequently, the crucible was cooled down to 23 °C, and the sample was tested. The time–temperature curve of the embedding process can be seen in Figure 3. 

The single-fiber pull-out tests were carried out in a standard climate (23 °C and 50% r. H.) on five specimens at a speed of 5 mm/min using the FAVIMAT+ from Textechno Herbert Stein GmbH & Co. KG (Moenchengladbach, Germany). The fiber end, which was not embedded, was fixed in the clamp, while the crucible was fixed in a sample holder above (as shown in Figure 2). The distance in between is described as the free fiber length. As the clamp moved down, the fiber was pulled out of the matrix, and the FAVIMAT+ recorded the load–displacement curves. The displacement was registered by the crosshead travel. The displacement value can then be regarded as *l_ef_* and put into Equation (1).

### 2.8. Tensile Test 

The 1A test specimens according to DIN EN ISO 527 were characterized after at least 48 h in a standard climate at 23 °C and 50% r. H. using a UPM 1446 testing machine from ZwickRoell GmbH & Co. KG (Ulm, Germany). Tensile tests were carried out according to DIN EN ISO 527 at a speed of 5 mm/min (fiber-reinforced) and 25 mm/min (non-reinforced material). The Young’s modulus, the tensile strength, and the average elongation or elongation at break of the test specimens were evaluated. Due to high elongation, the non-reinforced specimens were only pulled until 100% strain. The number of tested samples per composite was five.

### 2.9. Notched Impact Test 

Instrumented notched (notch A, DIN EN ISO 179-2) impact tests were carried out on ten test specimens per composite in the standard conditions of 23 °C and 50% r. H. The specimens were notched using the NOTCHVIS notching machine from CEAST/ INSTRON (Darmstadt, Germany). A Zwick GmbH & Co. KG (Ulm, Germany) Charpy impact machine and a 5 J pendulum was used. The impact, fracture and residual work, as well as the notched impact strength were recorded.

### 2.10. Scanning Electron Microscopy (SEM) 

The fracture surface of the fiber-reinforced specimens, caused by the tensile test, were observed with the help of SEM examinations. For this purpose, the MV2300 from CamScan Electron Optics Services (Ottawa, ON, Canada) was used. With this optical examination method, a prediction about the qualitative fiber-matrix adhesion could be made. An acceleration voltage of 10 kW and magnifications of 25×, 500×, and 1000× were used. To enable the SEM to scan the test specimens, the fracture surfaces were sputter-coated with gold before observation.

### 2.11. Contact Angle Measurement 

The free surface tension measurements were performed on aged (T, H, W) non-reinforced PA5.10 using the DSA 20B contact angle measurement test device from Krüss GmbH (Hamburg, Germany). The surface polarity of the material has a great influence on the fiber-matrix adhesion. For this reason, this investigation should be considered when interpreting the results. The measurements were conducted with water and diiodo-methane to determine the disperse and polar fractions. Ten repetitions of each fluid were performed per material composite.

## 3. Results and Discussion

### 3.1. Fiber Length Distribution, Surface Morphology, and Tension 

**Figure 4:**
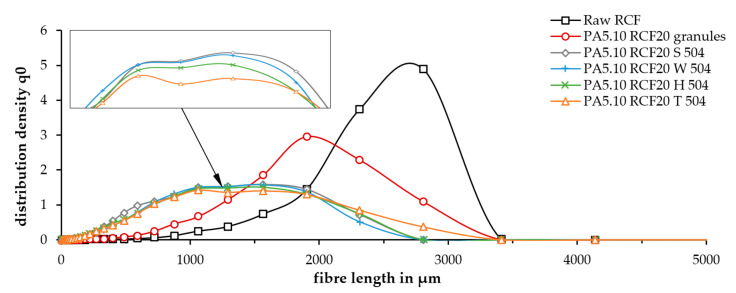
Fiber length distribution of raw RCF, compounded PA5.10 RCF granules, and parts of the accelerated-aged test specimen (taken from the parallel section).

The great differences in the fiber lengths during the processing steps can be seen in Figure 4. The raw fiber had a narrow distribution function and a mean fiber length of 2.8 mm, which is even longer than stated in the manufacturer’s data (2.5 mm). Following the compounding process, the mean length decreased to 1.9 mm and the distribution function widened and flattened. The injection molding process reduced the fiber length, on average, to 1.4 mm, which was about half of the initial length. In addition, it can be observed that the fiber lengths scattered strongly from 0 mm to over 2.5 mm, which is visible with the very flat and broad distribution.

The mean values of the fiber lengths can be seen in Table 4. Zarges et al. [32,33] already showed that, apart from the influence of the increased temperature, the highest impact on the RCFs in the processing was the shear force. For this reason, the immense shortening of the fiber after the compounding and injection molding process can be explained by the mechanical impact. In contrast to this shortening, among all four accelerated-aged test specimens, only slight differences can be seen. These differences are, as expected, all within the standard deviation since no further shearing forces were introduced to the material by aging. It can be assumed that the differences were caused by the standard deviation of the device and not by aging.

**Figure 5:**
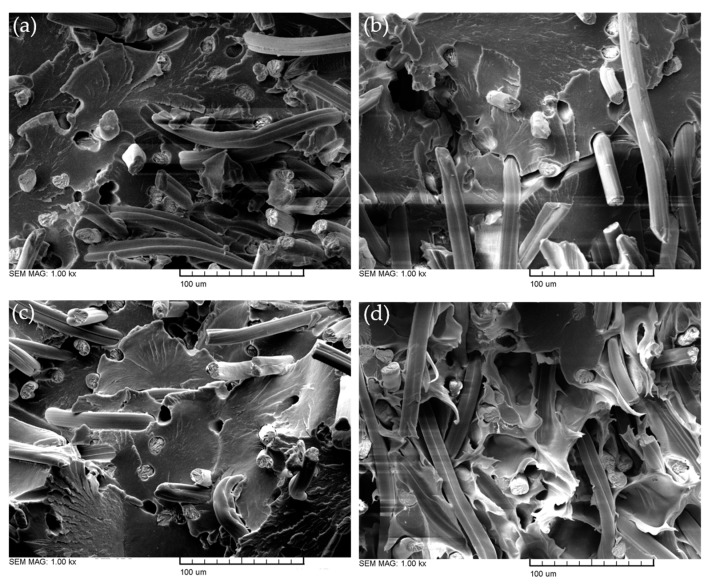
SEM pictures of PA5.10 RCF20 S504 (**a**), T504 (**b**), H504 (**c**), and W504 (**d**).

The SEM examinations in Figure 5 show significant changes on the surface due to the different aging conditions. All four pictures were taken in the middle of the fracture surface of the tensile test specimens. Whilst the standard climate specimen (a) features a relatively smooth surface with visible fraction parabolas, the aged fracture surfaces show different characteristics. The temperature storage specimen (b) has deep cracks on the entire surface due to the accelerated hydrolysis processes, while the humidity sample (c) shows scale-like matrix separations. Similar fibril-like separations were observed by Klein et al. [34] on cellulose acetate after aging. Apart from these findings, the water storage specimen (d) had a completely different surface morphology. The matrix had a ductile material behavior as it was not flat but tattered, which was caused by increased moisture absorption. Ksouri et al. [35] found a correlation between increased cracking in fiber-reinforced polyamide specimens due to storage in various high temperatures after increased water absorption in a water bath. Their water storage specimens also showed a rough surface with fibrils, which is an indication of a high plastic deformation prior to fracture. 

All four samples showed fiber pull-out holes and fiber breakage with distance as well as directly on the surface. As Zarges et al. [36] and Kahl et al. [37] have already observed, the fibers stuck straight out of the surface. That indicates that the fiber orientation proceeds in the direction of loading during the injection molding process. This enhances the mechanical properties, as the fibers can take the tension force at a nearly 0° angle, as Zarges et al. [38] verified in their paper. In contrast to the results of Harrass et al. [39], who investigated glass fiber-reinforced polyamides, there was no matrix coverage on the RCF after the pull-out, neither before nor after the aging in a water storage. As no matrix remained on the fiber, it could not increase the friction through adhesion under tensile load. This indicates low fiber-matrix adhesion in general. 

**Figure 6:**
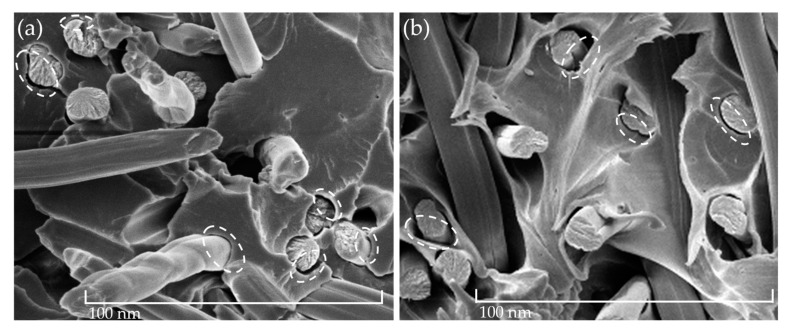
Detailed view of fiber-matrix debonding of PA5.10 RCF T504 (**a**) and W504 (**b**).

In addition to the SEM pictures shown in Figure 5, the fiber-matrix adhesion after climate storage was qualitatively analyzed. In Figure 6, it can be seen that there was weak fiber-matrix adhesion due to the gaps that emerged between the RCF and the matrix. This phenomenon appeared after all three accelerated aging scenarios and can be seen exemplary in Figure 6 after the high temperature (a) and water storage (b). Ksouri et al. [35], Gemmeke at al. [40], and Kahl et al. [41] have also observed the matrix debonding after aging using high temperature and water. Arif et al. [42] proved in their study that microcracks and gaps in the fiber-matrix bond have a strong dependency on the relative humidity. The debonding accelerates with a high water uptake and affects the mechanical properties negatively [42].

**Figure 7:**
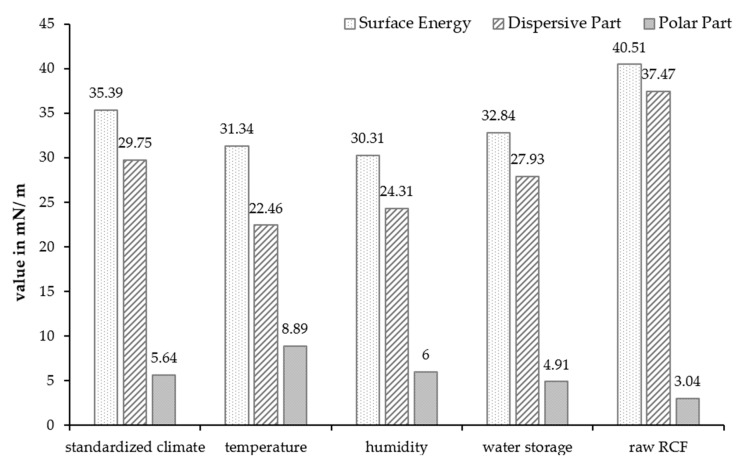
Results of the contact angle measurements of the accelerated-aged and non-reinforced PA5.10 in comparison with the surface energy of raw RCFs [43].

The results of the contact angle measurements of the non-reinforced PA5.10 are shown in Figure 7. For better comparability, the results of Kahl et al. [43] regarding the surface energy of raw RCFs were added on the right side. The figure shows that the total surface energy of the PA5.10 decreased after the accelerated aging, but mostly with the high-humidity aging. The polar fraction increased after the high-temperature aging. After the water storage, the polar part decreased at about 13%. Figure 7 shows an increase in the polar part of the temperature- and humidity-aged samples. The increased moisture content resulted in a reduced polar part of the water storage specimens [22]. Compared to the RCFs, the total surface energy of the PA5.10 was much lower but the polar part was elevated.

**Figure 8:**
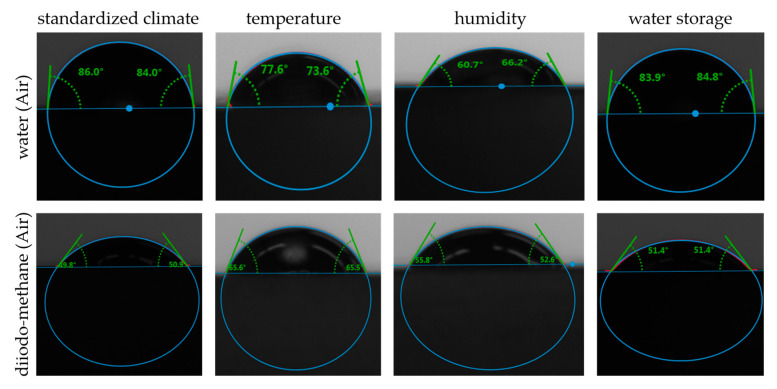
Water and diiodo-methane droplets of the contact angle measurements.

The droplets resulting from the contact angle measurements were used to calculate the surface energy. Characteristic drops of each artificially aged specimen can be seen in Figure 8 below. The contact angles varied due to the aging. The diido-methane droplets were flatter than the water drops, as the predominantly non-polar liquid had a lower surface tension than water. However, it still formed easily measurable contact angles.

### 3.2. Single-Fiber Tensile and Pull-Out Test

**Figure 9:**
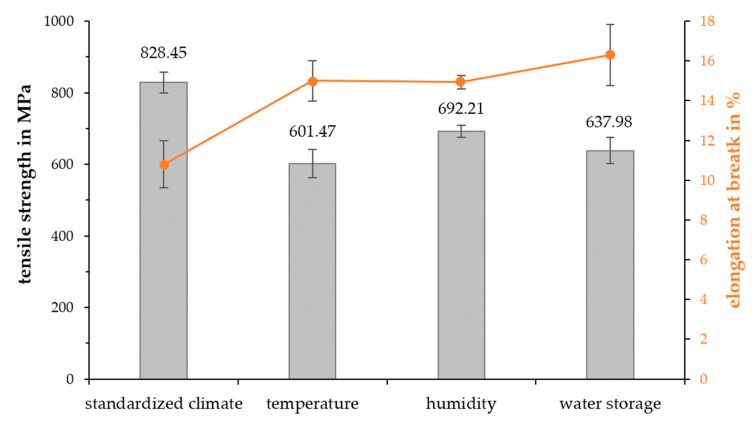
Single-fiber tensile strength of the aged RCF.

The results of the SFTT with the accelerated-aged RCFs are shown in in Figure 9. With 828.45 MPa, the tensile strength of the RCFs in the standard climate corresponded approximately to the value of the data sheet (830 MPa) from Cordenka GmbH & Co. KG (Obernburg am Main, Germany). As a consequence of the accelerated aging, the tensile strength of the RCFs was highly decreased. The value of the high-temperature-aged RCFs decreased by about 30%, while the water-stored fibers show a decrease of 23%, and the high-humidity-aged fibers show a 16% decrease in tensile strength. In contrast, the elongation at break of the RCFs increased by up to 40% after the accelerated aging. 

The property changes of the humidity and water storage samples were due to water absorption. Tatsuko et al. [44] and Zarges [45] have shown that the tensile strength of RCFs decreases with water absorption. This can be explained by the structural changes in the amorphous regions due to moisture absorption and the associated bonding of the water molecules to the hydroxyl groups. There is an expansion of the amorphous areas due to the water absorption, which leads to the breakage of the hydrogen bonds in the crystalline areas. This phenomenon also leads to a higher elongation at break. The mechanical changes in the temperature-aged RCFs resulted from the thermal degradation. The splitting of the molecules by the high temperatures also led to a lower tensile strength and higher elongation at break, as Kahl et al. [45] also observed in their study.

The SFTT showed a huge decrease in the tensile strength of the aged fibers. This also affected the mechanical properties of the fiber-reinforced composites. Although the fibers were normally completely enclosed by the matrix, the ageing of the raw fibers shows a trend on the fiber aging and the impact of high temperature and humidity as well as a water bath on the cellulose. It is commonly known that cellulose fibers do not resist high temperatures above 200 °C, but even at 70 °C, the mechanical properties dropped significantly. This degradation after the aging was also the reason why no single-fiber pull-outs could be recorded with the aged fibers. However, even if the fibers in the composites were not affected as much by the accelerated aging as the raw fibers were, the impact on the tensile and impact strength are shown in this paper. 

**Figure 10:**
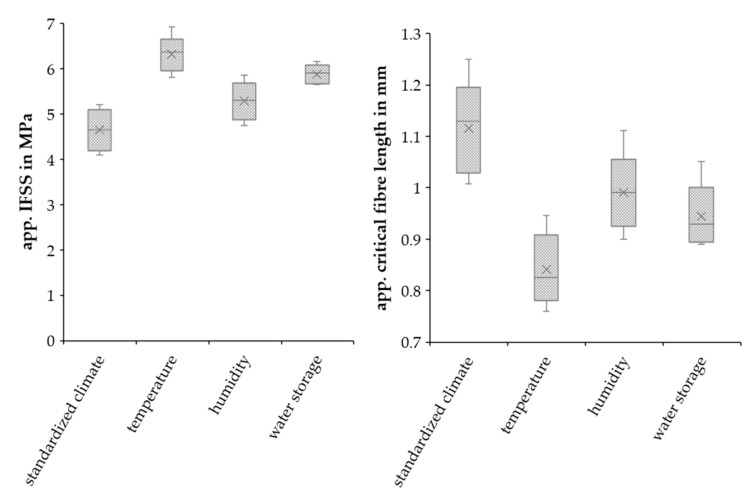
Apparent IFFS and critical fiber length of the RCFs with aged matrix.

Figure 10 presents the results of the SFPT with the raw RCFs and accelerated-aged PA5.10. The apparent critical fiber length (*l_c_*) and apparent IFSS were calculated using Equations (1) and (2). The higher the interfacial shear strength, the greater the adhesion between the fiber and matrix. The temperature aging featured the highest IFSS, with a value 36.8% higher compared to the reference. This was followed by the water storage, with an increase of 26.9%, and the humidity aging, with 13.9%. 

Consequently, it is possible to say that the aging of the matrix enhanced the fiber-matrix adhesion when tested with the SFPT together with unaged raw fibers. The enhancement of the temperature and humidity specimens can be explained by the increased polarity shown in Figure 7 [46]. The fiber-matrix adhesion of the water storage samples also increased, although the polarity of the composite fell. The enhancement cannot be explained by the tests performed in this study, but additional gel permeation chromatography (GPC) measurements are planned to examine whether chain cleavage by hydrolysis has an influence on the fiber-matrix adhesion. These results are different from the visual impression of the SEM pictures. This is because of the different modes of action of the fiber-reinforced specimens and the non-reinforced samples in the SFPT. Swelling, shrinkage, and moisture absorption decreased the fiber-matrix adhesion of the RCF-reinforced specimens. However, these effects did not have an impact on the non-reinforced samples. 

The critical fiber length is directly mathematically connected with the IFSS. By separating the fiber from the matrix using the SFPT a conclusion can be drawn on if and how the aged material effected the fiber-matrix adhesion. Figure 10 shows that the apparent critical fiber length of the unaged RCFs decreased after the climate storages. Following the high-humidity aging, the l_c_ decreased by about 12.3%, and after the water storage, by about 21.2%. The minimal apparent critical fiber length occurred after the high-temperature aging, with a value 26.9% lower in comparison to the standard climate specimens. If the critical fiber length was exceeded, the fibers failed only after they reached their maximum strength and could therefore optimally reinforce the matrix. Therefore, a longer fiber is needed to achieve maximum reinforcement. 

**Figure 11:**
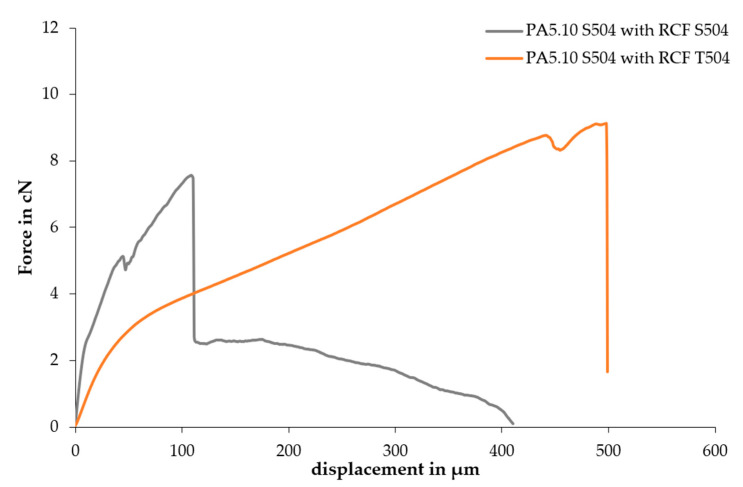
Force–displacement curves of the SFPT with PA5.10 S504 combined with the RCFs in a standard climate and after the temperature aging.

Additional tests were performed using PA5.10 at standard climate and 504 hours aged fibres (T, H, W), but the fibers always broke under the influence of the tensile force. In Figure 11, the force–displacement curves of the SFPT with PA5.10 S504 combined with RCFs in a standard climate and with RCFs after the temperature aging are shown. The subsequent path of the grey curve after the first drop corresponds to the pulling out of the fiber. This path cannot be seen in the results of the SFPT with the temperature-aged fibers. From this, it can be deduced that the RCFs broke before their pull-out could be recorded. The longer displacement of the aged fiber pull-out can be explained by the increased elongation at break after aging, as seen in Figure 9. Therefore, the pulling out of the fiber was not possible after the temperature, humidity, and water storage aging. The breakage can be explained by the reduction in fiber strength (Figure 9). For this reason, there were no additional combined tests with the aged fiber and aged matrix planned. 

### 3.3. Mechanical Property Analysis

**Figure 12:**
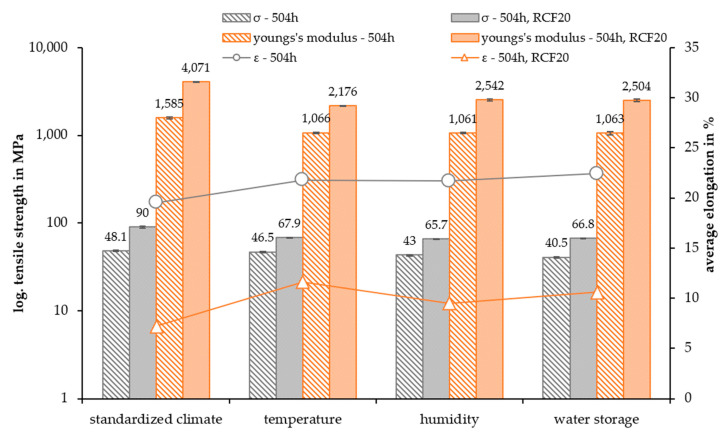
Tensile strength, Young’s modulus, and average elongation of the accelerated-aged test specimen after 504 h.

The tensile strength, Young’s modulus, and the average elongation of the non-reinforced and 20 wt.% fiber-reinforced PA5.10 are shown in Figure 12. The results of the tensile tests show the expected higher tensile strength of the 20 wt.% RCF-reinforced composites. In comparison to the reference data, the values decreased due to the accelerated aging. In general, the fiber-reinforced composites showed a higher decrease in the tensile strength after 504 h, especially in comparison to those in the standard climate. Arif et al. [42] showed that the relative humidity strongly impacted the damage mechanisms of fiber-reinforced composites. A higher relative humidity resulted in a higher damage level. The predominant damage mechanisms were fiber-matrix debonding at the fiber ends and surfaces and fiber breakages. In addition, there was brittle matrix crack propagation accompanied by a locally strained matrix zone around the debonded fibers, where visible and ductile matrix microcracks occurred [42]. 

The Young’s modulus of the RCF-reinforced composites resulted in a Young’s modulus more than 200% higher than that of the non-reinforced material, as Mamun et al. [16] and Gemmeke et al. [40] have also observed. The value of the fiber-reinforced composites highly decreased because of the accelerated aging. However, the aging did not have a huge impact on the non-reinforced specimens. The decrease in the RCF-reinforced modulus was much larger due to the aging compared to the raw material ones. A decreasing Young´s modulus because of moisture absorption due to high humidity or because of increased temperatures have also been seen in the studies by Arif et al. [42] and Basso et al. [10]. Both studies have shown a correlation of cracks and damages with aging and the fracture behavior, resulting in a lower Young’s modulus and tensile strength. 

Furthermore, the tensile strength was dependent on the fiber-matrix bond and the fiber length. The SEM pictures show a gap between both components (Figure 6), which has also appeared in the studies of Gemmeke at al. [40] and Kahl et al. [41]. Due to swelling and subsequent shrinkage of the matrix, the fibers detached from the matrix, which resulted in a poor fiber-matrix adhesion. This phenomenon is very visible in the tensile test results of the fiber-reinforced water storage specimens. 

The average elongation of the test specimen, as expected, decreased by about 10–13% with the fiber reinforcement. Furthermore, the elongation increased after the temperature, humidity, and water storage. In addition, the non-reinforced test specimens also show decreased tensile and notched impact strength. This supports the assumption of material degradation due to hydrolysis processes, which, therefore, has a significant impact on the fiber-matrix adhesion as well. 

In general, the accelerated aging had a significant impact on the mechanical and optical properties and on the fiber-matrix adhesion of the fiber-reinforced bio-polyamide. The tensile strength of the accelerated-aged specimen, in particular, the fiber-reinforced composites, decreased significantly with a slightly higher elongation at break. The increase in the elongation was already discussed by Maϊza et al. [47] for a polyamide 11. This can be explained by the fact that the SEM pictures show changes in the matrix. As a result of additional humidity in the material, which enhances hydrolysis processes [23], the ductility of the bio-polyamide increased due to the humidity aging and the water storage. Furthermore, there were a lot of cracks in the temperature-aged specimens due to accelerated hydrolysis, which reduced the strength of the compound due to a significantly higher crack initiation. In addition to the decreasing fiber length, this also had an impact on the Young´s modulus, which also decreased by a lot after the temperature aging [48]. 

**Figure 13:**
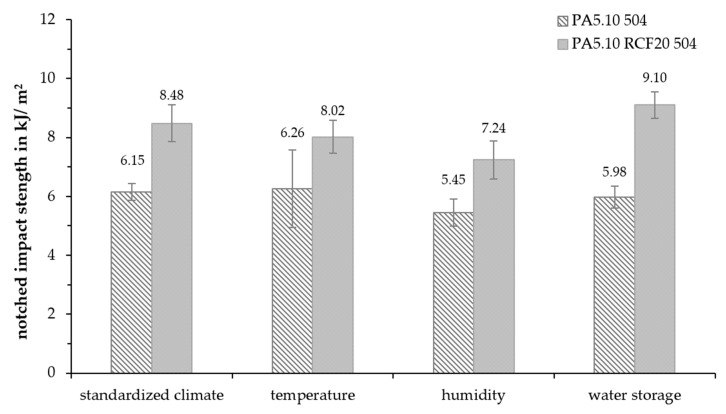
Notched impact strength of the accelerated-aged test specimens after 504 h.

In Figure 13, the results of the notched impact tests are presented. It is noticeable that the notched impact strength increased by 30–40% with the RCF reinforcement. Due to the high-temperature and humidity aging, the notched impact strength slightly decreased, but after the water storage, the value increased slightly. However, the RCF-reinforced specimens were affected more than the non-reinforced samples. 

Zarges et al. showed that the energy in a fiber pull-out is greater than in a fracture. This absorbed energy is significant for the notched impact strength [13,20,21]. Additionally, the matrix also showed an opposite impact on the value. The notched impact strength increased slightly due to the increased ductility of the water storage fiber-reinforced specimens due to water absorption. This was also seen by Thomason et al. [21]. Therefore, there is a correlation between the increased ductility after the accelerated aging and the impact strength. In addition, the decreased single-fiber tensile strength also deteriorated the impact strength. In particular, the temperature-aged RCFs had a reduced tensile strength, which led to fiber breakage at a much lower force than the S504 fibers.

## 4. Conclusions

Due to the correlation of all the findings elaborated in this study, the results are jointly considered at this point. In general, the results of this study show that accelerated aging with high temperatures, humidity, and water storage had a significant influence on the fiber-matrix adhesion of RCFs in PA5.10. Moreover, tensile tests revealed that the accelerated aging affected the stress–strain response of the composites. This was mainly caused by the regenerated cellulose fiber debonding from the matrix, which lowered the fiber-matrix adhesion. This led to a lower tensile strength of the fiber-reinforced composites. The notched impact strength of the RCF-reinforced composites decreased after the accelerated aging because of the decreased single-fiber tensile strength of the accelerated-aged RCF. 

SEM examinations showed an even distribution between the fiber pull-out holes and the broken fibers. Therefore, the specimens did not only rupture under force due to a bad fiber-matrix adhesion, which causes the fiber pull-outs, but because of an even balance of fiber breakage and the fiber-matrix bond. Based on the results of the SFPT, the results of the tensile tests can be discussed in depth. The IFSS increased after the accelerated aging. This lies in contrast with the SEM pictures, which show decreasing tensile strength, and the results of the SFTT. The different modes of action of the aged fiber-reinforced specimens and the aged non-reinforced samples in the SFPT resulted in a different material behavior. Due to swelling, shrinkage, and moisture absorption, the matrix debonded from the fibers, which lowered the fiber-matrix adhesion of the RCF-reinforced specimen. However, these effects did not have an impact on the non-reinforced samples as in the SFPT; the aged matrix melted and enclosed the fiber completely. This procedure set up and restored the fiber-matrix adhesion, and therefore, the plain influence of the polarity of the material on the fiber-matrix adhesion can be observed. Kahl et al. [43] proved that the free surface tension of RCFs has a significant impact on the fiber-matrix adhesion. For this reason, the disperse and polar fractions of the accelerated-aged composites were considered. The polarity of the non-reinforced PA5.10 was enhanced due to the high-humidity and -temperature aging, However, due to the matrix-debonding of the fibers in the RCF-reinforced composites, this factor did not affect the fiber-matrix adhesion significantly, as shown in the mechanical properties of the accelerated-aged test specimens. The critical fiber length, which also resulted from the SFPT, decreased due to a better fiber-matrix adhesion. 

These results of this study prove the damaging effect on RCFs due to artificial aging and the influence on the mechanical properties of fiber-reinforced bio-based PA composites. Up until now, not much has been known about the persistence of bio-PA in this field of research, but the existing literature is consistent with the results found in this study. The research adds value to the research on bio-based composite alternatives, for example, for the automotive sector, where petro-based polyamide is a highly used polymer.

The main findings of the study are listed below:

- The RCFs are less resistant against accelerated aging than the bio-polyamide, which was shown in single-fiber tests.
- Single-fiber pull-out tests showed an increase in the fiber-matrix adhesion due to the different storage climates, which could be set in relation to the increasing polarity of the aged bio-polyamide.
- The accelerated aging led to a debonding of the fiber from the matrix because of swelling and shrinkage of the fiber and matrix as well as moisture absorption, which had a significant impact on the fiber-matrix adhesion and the mechanical properties.
- The Young’s modulus strongly decreased after the accelerated aging in all non- and RCF-reinforced composites.
- The accelerated aging enhanced the elongation at break but decreased the tensile strength of all composites.

## Figures and Tables

**Table 1:** Composite designations and their accelerated aging.

Composite Name	Regenerated Cellulose Fibers in wt.%	Accelerated Aging	Storage Time in Hours	Temperature in °C	Relative Humidity in%
PA5.10 S504	-	Standard climate	504	23	50
PA5.10 H504	-	High humidity	504	23	90
PA5.10 T504	-	High temperature	504	70	50
PA5.10 W504	-	Water storage	504	23	-
PA5.10 RCF20 S504	20	Standard climate	504	23	50
PA5.10 RCF20 H504	20	High humidity	504	23	90
PA5.10 RCF20 T504	20	High temperature	504	70	50
PA5.10 RCF20 W504	20	Water storage	504	23	-

Reference values in orange.

**Table 2:** Temperature profile (setpoints) of the twin-screw extruder.

Zone	1	2	3	4	5	6	7	Nozzle
Temperature in °C	230	220	220	215	215	210	210	215

**Table 3:** Temperature profile of the injection molding process.

Zone		1	2	3	4	5	Nozzle	Mold Temperature
Temperature in °C	PA5.10	80	240	245	250	255	260	40
	PA5.10 RCF20	80	220	230	230	240	240	40

**Table 4:** Mean values of the fiber lengths of all composites.

Composite Name	Mean Value of the Fiber Length in mm
Raw RCF	2.808
PA5.10 RCF20 granules	1.903
PA5.10 RCF20 S504	1.566
PA5.10 RCF20 T504	1.263
PA5.10 RCF20 H504	1.498
PA5.10 RCF20 W504	1.530

## Data Availability

The data presented in this study are available upon request from the corresponding author.
